# A Supervised-Reinforced Successive Training Framework for a Fuzzy Inference System and Its Application in Robotic Odor Source Searching

**DOI:** 10.3389/fnbot.2022.914706

**Published:** 2022-05-31

**Authors:** Xinxing Chen, Yuquan Leng, Chenglong Fu

**Affiliations:** ^1^Shenzhen Key Laboratory of Biomimetic Robotics and Intelligent Systems, Shenzhen, China; ^2^Guangdong Provincial Key Laboratory of Human-Augmentation and Rehabilitation Robotics in Universities, Southern University of Science and Technology, Shenzhen, China

**Keywords:** supervised learner, reinforcement learning, fuzzy inference system, robotic odor source searching, Monte Carlo test

## Abstract

Fuzzy inference systems have been widely applied in robotic control. Previous studies proposed various methods to tune the fuzzy rules and the parameters of the membership functions (MFs). Training the systems with only supervised learning requires a large amount of input-output data, and the performance of the trained system is confined by that of the target system. Training the systems with only reinforcement learning (RL) does not require prior knowledge but is time-consuming, and the initialization of the system remains a problem. In this paper, a supervised-reinforced successive training framework is proposed for a multi-continuous-output fuzzy inference system (MCOFIS). The parameters of the fuzzy inference system are first tuned by a limited number of input-output data from an existing controller with supervised training and then are utilized to initialize the system in the reinforcement training stage. The proposed framework is applied in a robotic odor source searching task and the evaluation results demonstrate that the performance of the fuzzy inference system trained by the successive framework is superior to the systems trained by only supervised learning or RL. The system trained by the proposed framework can achieve around a 10% higher success rate compared to the systems trained by only supervised learning or RL.

## 1. Introduction

Fuzzy inference systems have been applied in various classification and regression problems in machine learning (Nguyen et al., 2019; Wu et al., 2019; Cui et al., 2020) and have also been widely used in control and optimization in robotics (Chen C. et al., 2022; Su et al., 2022). Previous studies have proposed several methods to learn and tune the fuzzy rules and the parameters of the membership functions (MFs) to achieve the expected performance. Some widely applied methods design the fuzzy systems from (1) a manually-built fuzzy rule look-up table (Chen and Huang, 2020b); (2) learning from collected input-output data (Wang et al., 2020) through evolutionary algorithms (Wu and Tan, 2006) and gradient descent (Wang and Mendel, 1992).

Unfortunately, in unknown environments, prior knowledge may not be sufficient to build well-designed fuzzy rules, and the parameters of the system can hardly be tuned to an optimal solution (Dai et al., 2005). In terms of learning from collected data, a typical work is that Wang and Pang (2020) proposed to train adaptive neural fuzzy inference systems (ANFIS) to mimic existing bio-inspired controllers and probabilistic controllers for odor source searching utilizing collected input-output data and realize behavior patterns similar to the target controller. The performance of the trained fuzzy inference system-based controller can be further improved by fusing the input-output data of two different controllers.

The above learning process is in the scope of supervised training, in which a large amount of training data is required. Data collection can be time-consuming and the data collected from limited environmental settings may not include boundary conditions. In addition, for some complex environments, existing controllers may not be optimal. Learning from them cannot necessarily achieve the desired performance.

Reinforcement learning (RL) has attracted researchers' attention in the past decades because it provides an effective solution to robotic control and decision-making problems for which analytically optimal solutions are hard to obtain. RL is based on a human-inspired “trial-and-error” learning process that action will be reinforced if it is followed by a desired state of the robot. Since RL can tune the controllers in real-time, correct action or trajectory data is not required. Therefore, RL is especially suitable to operate in a knowledge-poor environment.

In previous studies, the fuzzy inference system has been integrated into RL in various application scenarios because of its high interpretability and flexibility. Kumar et al. (2020) used a fuzzy inference system to switch between three working modes for the traffic light control system, while a deep RL model was designed to switch the traffic lights. The fuzzy inference system and the RL model worked in a hierarchical framework. Wang et al. (2021) integrated a fuzzy inference system into the reward function of the RL model to balance the exploitation and exploration during odor source searching. Er and Deng (2004) proposed a fuzzy Q learning method to tune a fuzzy inference system-based actor model by RL, and similar methods have been applied in autonomous vehicle control (Dai et al., 2005) and robotic odor source searching (Chen and Huang, 2019; Chen X. et al., 2022).

Previous studies usually initialized the parameter of the fuzzy inference system with conventional clustering methods (Cui et al., 2020; Wang et al., 2020) or arbitrarily manual settings. Although RL can tune a fuzzy inference system to achieve a good performance, it remains an interesting problem to investigate whether the initial parameter setting of the fuzzy inference system will affect the performance of the system after numerous training epochs. To the best of our knowledge, no previous studies focused on this problem and provided a good solution to initialize the fuzzy inference system so that it can achieve better performance after training.

In this paper, a supervised-reinforced successive training framework for a multi-continuous-output fuzzy inference system (MCOFIS) was proposed. In this framework, the MCOFIS was first trained with input-output data from an existing state-action model. The input-output data was collected in multiple robotic tasks, in which the robot was running a pre-designed controller. The measured state of the environment and the resulting actions of the robot at each time step were recorded as the input-output data. After this supervised training stage, the trained MCOFIS model was utilized as the initial model in the process of reinforcement training and further trained with the deep deterministic policy gradient (DDPG) RL algorithm (Lillicrap et al., 2015). The proposed training framework was applied in a robotic odor source searching problem, which was usually solved by bio-inspired reactive algorithms (Shigaki et al., 2019), probabilistic algorithms (Vergassola et al., 2007; Chen and Huang, 2020a; Chen et al., 2020), and learning algorithms (Wang and Pang, 2020; Chen et al., 2021a) in previous studies. The performance of the trained MCOFIS-based odor source searching controller was compared with the MCOFIS-based controller trained with RL only. The results showed that the MCOFIS trained with the proposed successive framework can promote the success rate of odor source searching to around 95%, while the success rate of the model trained with only RL was around 85%.

The rest of the paper is organized as follows: Section 2 presents the structure of the MCOTSK model, how the successive training framework is utilized to tune the system, and the application of the proposed method in odor source searching. Section 3 compares the controller trained with the proposed method and the controller trained with only supervised training or reinforcement training and analyzes the results. Section 4 presents some discussions. Section 5 concludes the paper.

## 2. Methods

**Figure 1:**
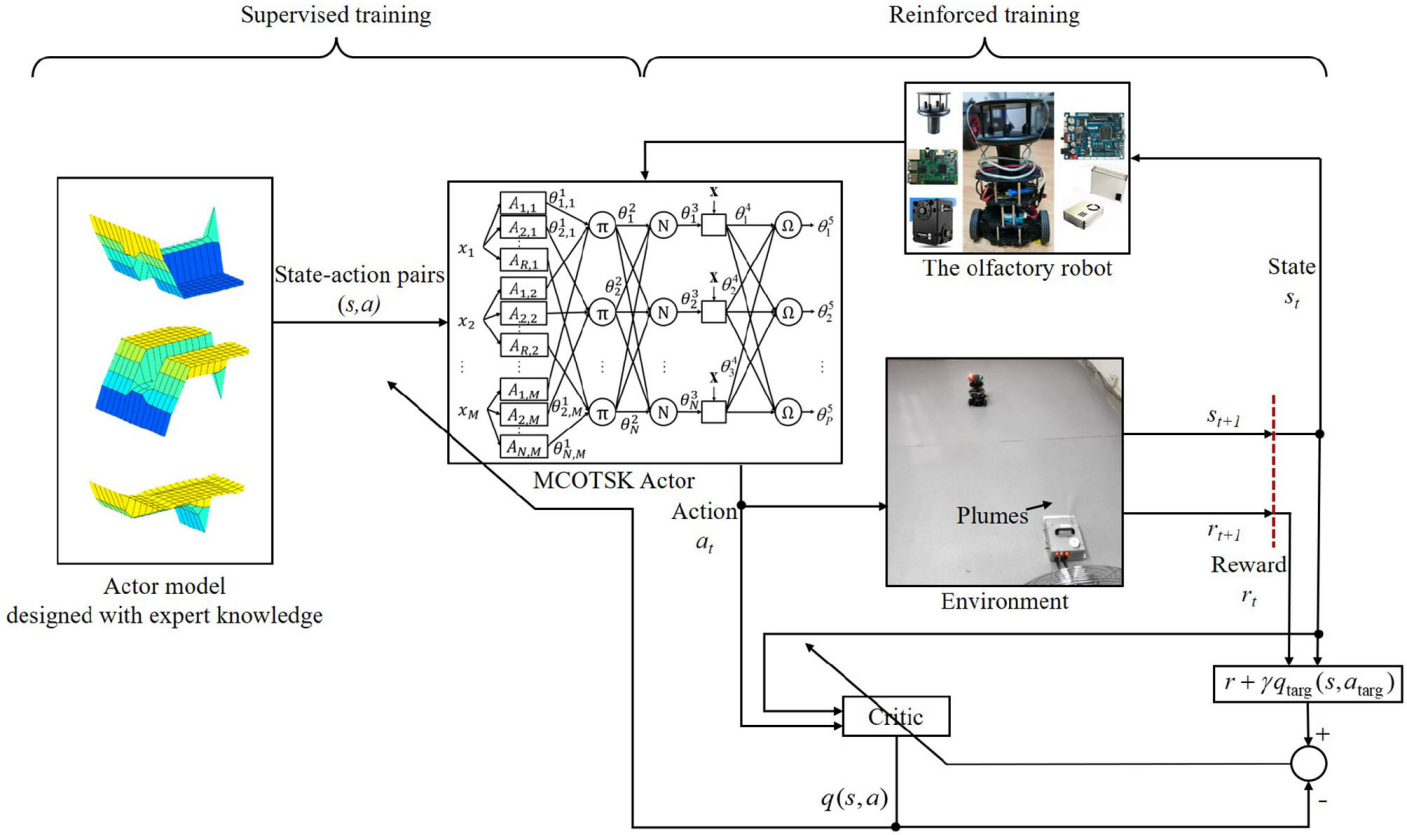
Illustration of the supervised-reinforced successive training framework for the multi-continuous-output TSK fuzzy inference system (MCOTSK).

In this section, the proposed supervised-reinforced successive training framework for an MCOTSK is introduced. The MCOTSK serves as an “Actor” mapping the state *s* to the action *a* of the robot. The state means the observed state of the environment, which is measured by the sensing system of the robot. The action means estimated control commands for the robot. As illustrated in Figure 1, the proposed training framework consists of two parts: in the supervised training part, the MCOTSK is trained offline with numerous state-action pairs collected from robot-environment interactions when the robot is driven by a pre-designed controller; in the reinforced training part, the MCOTSK is trained online by maximizing the expected future cumulative reward when the robot's action is estimated by the MCOTSK Actor. The structure of the MCOTSK model and two successive training parts are introduced in the following subsections.

### 2.1. The Structure of the MCOTSK Model

The MCOTSK model is a variation of the general TSK fuzzy inference system (Chen X. et al., 2022). As depicted in Figure 1, the MCOTSK model consists of five layers, in which the adjustable nodes are represented by rectangles, and the fixed nodes are represented by circles. *A*_*n, m*_(*n* = 1, …, *N*; *m* = 1, …, *M*) are fuzzy sets.

Assuming the MCOTSK model has *M* inputs: *x*_1_, …, *x*_*M*_ ∈ **R**, the inputs are fuzzified by *N* fuzzy rules in the first layer, which is called the fuzzification layer. The outputs of this layer are formulated as follows:


(1) $$\begin{array}{l} \theta_{n,m}^{1}=\mu_{A_{n,m}}\left(x_{m}\right)=e^{-\frac{{\left(x_{m}-c_{n,m}\right)}^{2}}{2a_{n,m}^{2}}}, \end{array}$$

where μ_*A*_*n, m*__ represents the membership function of the fuzzy set *A*_*n, m*_(*n* = 1, …, *N*; *m* = 1, …, *M*) and is set to be a Gaussian membership function (MF) in this paper. *a*_*r, m*_ and *c*_*r, m*_ are hyper-parameters adjusting the distribution of the Gaussian MFs.

The section layer is a fixed layer, in which all the nodes are marked as π. The outputs are the firing level of the rules, and are formulated as follows:


(2) $$\begin{array}{l} \theta_{n}^{2}=\overset{M}{\underset{m=1}{\prod }}\mu_{A_{n,m}}\left(x_{m}\right)\;\left(n=1,\ldots ,N\right). \end{array}$$

The third layer is the normalization layer. It normalizes the outputs of the second layer to represent the contribution of the *n*th fuzzy rule to the sum of the firing level of all rules. The output of this layer can be expressed as follows:


(3) $$\begin{array}{l} \theta_{n}^{3}=\frac{\theta_{n}^{2}}{\sum_{k=1}^{N}\theta_{k}^{2}}. \end{array}$$

The fourth layer is an adaptive layer, of which the output is the product of the normalized firing level calculated by the third layer and a linear polynomial of the inputs of the MCOTSK model:


(4) $$\begin{array}{l} \theta_{n}^{4}=\theta_{n}^{3}y_{n}\left(x_{1},\ldots ,x_{M}\right)=\theta_{n}^{3}\left(b_{n,0}+\overset{M}{\underset{m=1}{\sum }}b_{n,m}x_{m}\right), \end{array}$$

where *y*_*n*_ is the linear polynomial of Rule *n*, and *b*_*n*, 0_ and *b*_*n, m*_ are adjustable weight parameters.

The last layer is the output layer. It calculates the weighted sum of $\theta_{n}^{4}$. Assuming the MCOTSK model has *P* outputs, they can be expressed as follows:


(5) $$\begin{array}{l} \theta_{p}^{5}=\overset{N}{\underset{n=1}{\sum }}\omega_{p,n}\theta_{n}^{4}, \end{array}$$

where ω_*p, n*_ are adaptive weight parameters (*p* = 1, …, *P*; *n* = 1, …*N*).

In order to make the MCOTSK model estimate optimal actions from the input states of the environment, the adaptive parameters *a*_*n, m*_, *b*_*n, m*_, *b*_*n*, 0_, *c*_*n, m*_, and ω_*p, n*_ (*n* = 1, …, *N*; *m* = 1, …, *M*; *p* = 1, …, *P*) need to be tuned.

### 2.2. The Supervised Training Part

In the supervised training part, the proposed MCOTSK model learns from an existing suboptimal Actor, which was designed with prior knowledge. By running a robotic task with the suboptimal Actor for multiple trails, the state of the environment and the action the robot takes can be recorded. Numerous collected state-action pairs are utilized as input-output samples to train the MCOTSK model.

The centers of the MFs *c*_*n, m*_ are initialized using a conventional K-means clustering method, which is the same as Cui et al. (2020) and Wang et al. (2020). The SDs of the MFs *a*_*n, m*_ are initialized to be 1.

At each training epoch, a batch of state-action pairs (*a*_*i*_, *s*_*i*_), (*i* = 1, …, *BS*_s_) (batch size *BS*_s_ = 32 in this paper) are randomly selected from all the collected samples to tune the parameters of MCOTSK by minimizing the mean squared error between the estimated actions and the collected actions:


(6) $$\begin{array}{l} \phi^{*}=\underset{\phi }{\arg\min}\overset{BS_{\text{s}}}{\underset{i=1}{\sum }}{\left[\text{MCOTSK}\left(s_{i}|\phi \right)-a_{i}\right]}^{2}, \end{array}$$

where $\phi^{*}={\left\{a_{n,m},b_{n,m},b_{n,0},c_{n,m},\omega_{p,n}\right\}}^{*}$ is the optimal parameter set for the supervised-trained MCOTSK model.

The training process will terminate when the recorded minimum mean squared error on the evaluation set keeps unchanged for 40 training epochs. The optimal MCOTSK model is further used as the initial model in the reinforced training part.

### 2.3. The Reinforced Training Part

In the reinforced training part, the DDPG RL algorithm (Lillicrap et al., 2015) is applied to further train the MCOTSK model through the “trial-and-error” process.

A “Target actor” is initialized the same as the MCOTSK Actor optimized in the supervised training part. A “Critic” model and its twin “Target critic” model are two artificial neural networks initialized with the same structure and parameters and serve as the action-value functions *q*(*s, a*) and *q*_targ_(*s, a*), which calculated the expected cumulative future reward of the current state-action pair.

At each step *t* during the robot's task, an action command *a*_*t*_ is estimated from the input state *s*_*t*_ with the MCOTSK model, and the robot takes the corresponding action. Then an updated state *s*_*t* + 1_ of the environment is perceived by the robot and serves as the input of MCOTSK at the next step. The experience of the robot (*s*_*t*_, *a*_*t*_, *r*_*t*_, *s*_*t* + 1_) is stored in an experience replay buffer D (buffer size = 5,000 in this paper). A batch of stored experience in D was randomly selected to tune the Actor and Critic model in each training epoch (batch size *BS*_r_ = 32 in this paper).

In a reinforced training epoch, *s*_*t* + 1_ is sent to the Target actor to estimate an action command *a*_targ, *t* + 1_ for the next state. The reward *r*_*t*_ the robot obtains at step *t* and the action value *q*_targ_ calculated with the Target critic were used to calculate the target action value *r* + γ*q*_targ_(*s*_*t* + 1_, *a*_targ, *t* + 1_). The Temporal-Difference error between the action value *q*(*s*_*t*_, *a*_*t*_) estimated by the Critic model and the target action value estimated by the Target critic model are used to optimize the Critic model by minimizing the following loss with stochastic gradient descent:


(7) $$\begin{array}{l} L(\phi_{\text{c}},D)=\underset{\left(s_{t},a_{t},r_{t},s_{t+1}\right)\sim D}{\text{E}}[(q\left(s_{t},a_{t}|\phi_{\text{c}}\right)\\ {-\left(r+\gamma q_{\text{targ}}\left(s_{t+1},a_{\text{targ},t+1}|\phi_{\text{c,targ}}\right)\right))}^{2}], \end{array}$$

where ϕ_c_ is the parameters of the Critic model, and ϕ_c, targ_ is the parameters of the Target critic model. The MCOTSK Actor is tuned by maximizing the estimated action value from the Critic model. Therefore, the loss function for gradient descent is set as follows:


(8) $$\begin{array}{l} \;L\left(\phi_{\text{a}},D\right)=-\underset{\left(s_{t},a_{t},r_{t},s_{t+1}\right)\sim D}{\text{E}}\left[q\left(s_{t},a_{t}|\phi_{\text{c}}\right)\right]\\ =-\underset{\left(s_{t},a_{t},r_{t},s_{t+1}\right)\sim D}{\text{E}}\left[q\left(s_{t},\text{MCOTSK}\left(s_{t}|\phi_{\text{a}}\right)|\phi_{\text{c}}\right)\right], \end{array}$$

where ϕ_a_ is the parameters of the Actor model, and ϕ_a, targ_ is the parameters of the Target actor model.

The parameters ϕ_a, targ_ and ϕ_c, targ_ are updated through a soft updating policy at each training epoch:


(9) $$\begin{array}{l} \phi_{\text{c,targ}}\leftarrow \rho \phi_{\text{c,targ}}+\left(1-\rho \right)\phi_{\text{c}}, \end{array}$$

(10) $$\begin{array}{l} \phi_{\text{a,targ}}\leftarrow \rho \phi_{\text{a,targ}}+\left(1-\rho \right)\phi_{\text{a}}, \end{array}$$

where ρ is 0.9 in this paper.

In order to reduce overfitting and increase generalization in training the MCOFIS, the DropRule technique (Wu et al., 2019) is applied in the training process. DropRule randomly drops some fuzzy rules (sets the firing level to zero) during the training process with probability *P* ∈ (0, 1) and remains the firing level unchanged with probability 1 − *P*. DropRule can promote the robustness of each individual rule. The Layer Normalization (LN) technique is used to normalize the firing level of the rules. The LN layer added in the MCOTSK model is expected to mitigate the gradient vanishing issues (Cui, 2022).

### 2.4. Application of the Training Framework in Odor Source Searching

In this paper, the proposed successive training framework is applied to an odor source searching problem to demonstrate its feasibility and superiority.

**Figure 2:**
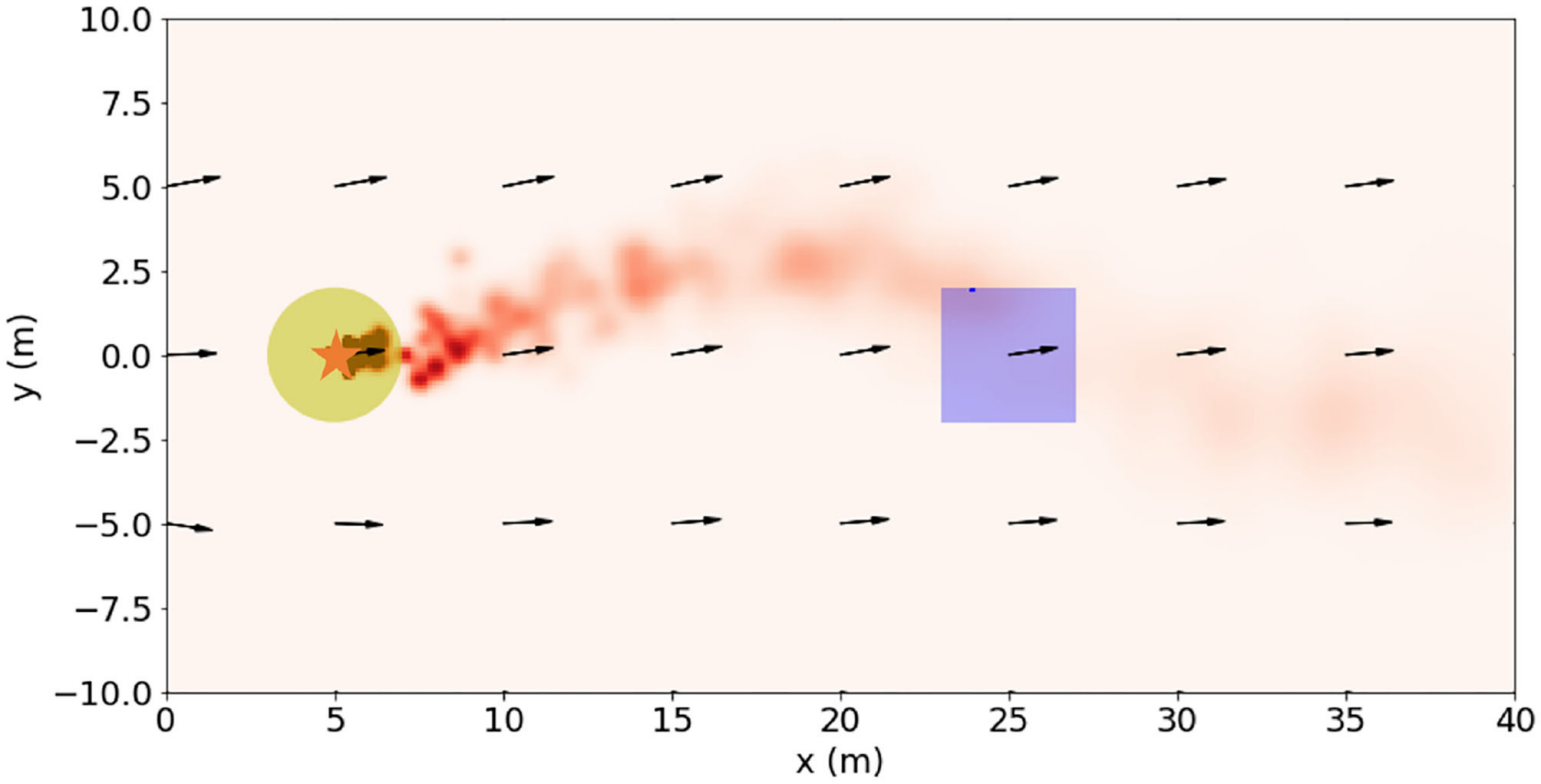
Illustration of a snapshot of the searching area in the odor source searching problem. The odor source is represented by the star. The yellow patches represent the area within 2 m from the odor source. The wind field is illustrated by the black arrows. The red puffs are the simulated odor plumes, which resemble the real-world plumes well as shown in Figure 1.

The odor source searching problem in this paper is defined as follows: in an outdoor environment in which the wind field is changing over time, the robot starts from a position away from the odor source and tracks dynamic odor plumes and reaches within 2*m* from the odor releasing source. The searching area is set to be 40*m* × 10*m*, and the coordinate system is shown in Figure 2. The odor leakage source can be regarded as a point and is located at (5, 0). The wind velocity is set as 1*m*/*s* in the searching space. The wind direction is aligned to X-axis at *t* = 0. The noise gain on the wind direction is 5. The odor plumes (illustrated as the red puffs in Figure 2) are released from the odor source and dispersed by the wind. The plumes are modeled by the filament-based odor plume dispersion model (Farrell et al., 2002) to simulate an intermittent 2D odor concentration distribution.

The robot runs a Lévy Taxis-based odor plume tracking algorithm, which is a variation of Fuzzy Lévy Taxis (Chen and Huang, 2020b), integrating the proposed MCOTSK Actor model. At each searching step, the robot turns its heading θ_a_ to an angle *T*_*a*_ and moves forward for a length *M*_*l*_. *T*_*a*_ and *M*_*l*_ follow the probability distribution presented in Equations (11), (12), and (13):


(11) $$\begin{array}{l} T_{a}=\left[2\cdot \arctan\left(\frac{1-\alpha }{1+\alpha }\tan\left(\pi \left(rnd-0.5\right)\right)\right)\right]+bias, \end{array}$$

(12) $$\begin{array}{l} M_{l}=L_{\min}\cdot rnd^{\frac{1}{1-\mu }}, \end{array}$$

(13) $$\begin{array}{l} bias=\beta \theta_{\text{u}}+\left(1-\beta \right)\theta_{\text{a}}. \end{array}$$

*rnd* is a random value uniformly distributed in [0, 1] and is resampled in each searching step. The key parameters α, β, and μ of the Fuzzy Lévy Taxis algorithm are determined by the proposed MCOTSK model. The inputs of MCOTSK are the states of the environment: the odor concentration *C*_*t*_ measured by the robot at its current position at time *t* and the concentration gradient ∇*C*_*t*_ = *C*_*t*_ − *C*_*t* − 1_. The outputs of MCOTSK go through a Tanh activation layer limiting the outputs in the range of [−1,1]. After a further rescale process, the range of the outputs can be adjusted suitable for the key parameters α, β, and μ. The rescaled outputs are the estimated action commands and are utilized to drive the robot.

**Figure 3:**
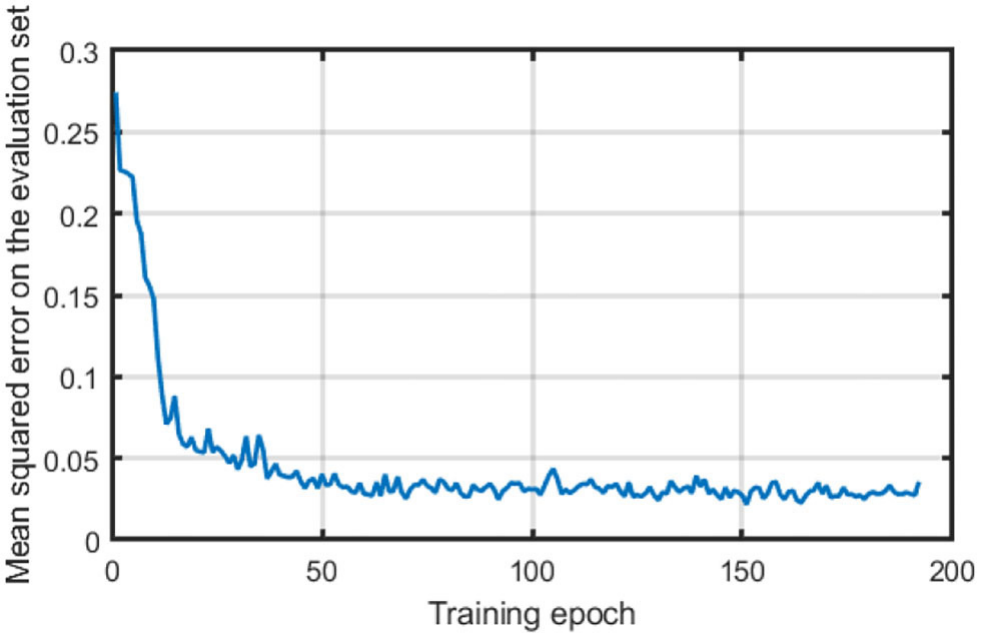
The mean squared errors on the evaluation set during the supervised training stage.

To apply the proposed successive training framework, 50 trails of odor source searching tasks are conducted, during which the robot runs the Fuzzy Lévy Taxis algorithm, and the state-action pair {*C*_*t*_, ∇*C*_*t*_, α, β, μ} is recorded at each time step *t*. A total number of 1,816 state-action pairs are collected in this study to train the MCOTSK model firstly with supervised learning. The learning rate of the supervised learning part is 0.01. The number of rules is set to be 10. The DropRule rate is 0.2. The mean squared errors on the evaluation set between the collected actions and the outputs of MCOTSK at each epoch are recorded during the supervised training and are shown in Figure 3.

**Figure 4:**
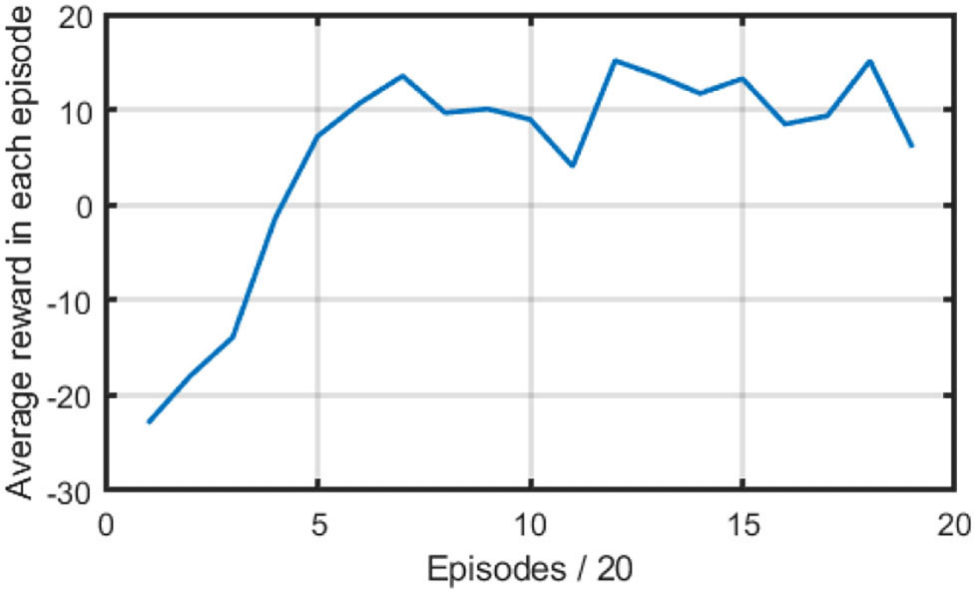
The average reward in each episode in the reinforced training stage.

The trained model is used as an initial model in the reinforced training stage. Every odor source searching task is a training episode. An episode will stop when the robots arrive within 2*m* from the odor source, exceeds the boundaries of the searching area, or the number of searching steps exceeds a limit, which is 60 steps in this paper. The learning rate of Actor is 0.0001 and that of Critic is 0.002. The reward of the robot obtained in step *t* is as follows:


(14) $$r_{t}\;=\;\{\begin{array}{ll} 20 & \text{if the robot arrives within 2 m}\\ & \text{ from the odor source,}\\ -10 & \text{the robot exceeds the boundaries}\\ & \text{ of the searching area,}\\ -1+\frac{C_{\text{c}}}{C_{0}}\cos(\theta_{\text{u}}-\theta_{\text{a}}) & \text{otherwise}. \end{array}$$

where *C*_0_ is a constant and set to 30 in this paper. This reward setting is designed to let the robot learn bio-inspired anemotaxis and chemotaxis behaviors. The models were trained for 360 episodes. During the process of training, we recorded the reward the robot obtained in each episode. Figure 4 presents the average reward for every 20 episodes during the reinforced training. It can be seen that the average reward started from around −22 because a large variation was added to the estimated action for exploration. With the added variation decayed, the average reward increased and converged to around 10. From the average reward curves, we can know that the robot can learn to track the dynamic plumes and find the odor source with the MCOTSK model trained by the proposed method.

## 3. Performance Evaluation

In order to demonstrate the advantages of the proposed training framework, Monte Carlo tests were conducted in a testing environment that is different from the training environment. The robots started from random positions in the rectangle area shown in Figure 2 and searched the odor source with 21 different action models—1: the Fuzzy Lévy Taxis algorithm used in the supervised training; 2 ~ 20: the trained MCOTSK model after every 20 reinforced training episodes (from 0 to 360 episodes); 21: the MCOTSK model trained with RL only. For each model, 200 trials were conducted.

**Figure 5:**
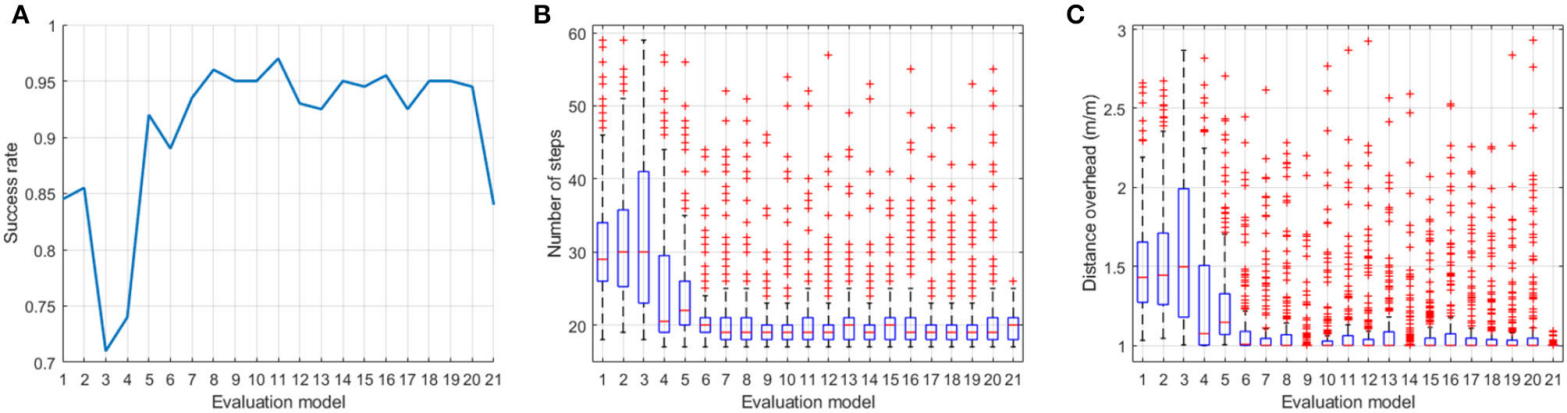
Performance evaluation results of the three controllers in the Monte Carlo tests. **(A)** Success rate, **(B)** is the number of searching steps, and **(C)** is the distance overhead. Model 1: the Fuzzy Lévy Taxis algorithm used in the supervised training; Models 2–20: the trained MCOTSK model after every 20 reinforced training episodes (from 0 episodes to 360 episodes); Model 21: the MCOTSK model trained with RL only.

The controllers were evaluated with three metrics. The first metric was the success rate: the proportion of trials in which the robot reached <2*m* from the odor source. The second metric was the number of searching steps in all successful trials. The third metric was the distance overhead, which is the traveled distance from the starting position to the stopping position divided by the straight distance in all successful trails. The latter two metrics reflect the efficiency of the searching process. The results of the Monte Carlo tests were shown in Figure 5.

It can be seen that the Fuzzy Lévy Taxis algorithm and the model trained with only the supervised stage can achieve a similar success rate (around 85%) and efficiency. It demonstrated that the MCOTSK has been trained to a suboptimal action model. When the reinforced training stage started, the success rate first decreased and then increased fast and exceeded 95%. The decrease at the early reinforce training stage is because the Critic model was being tuned. Once the Critic model can estimate the action value accurately, the performance of the MCOTSK-based Actor returned to the desired condition. With the proposed framework, the robot can learn some bio-inspired searching behaviors in the supervised training stage and fine-tune the parameters of the Actor model in the reinforcement training stage, which can avoid too much random parameter exploration and accelerate the reinforced training process. The performance of the trained model can also benefit from the pre-designed controller because it can provide correct guidance for the robot at the early stage during RL and serve as a baseline behavior pattern. Therefore, the model trained by the proposed framework can achieve better results compared with the model trained by RL only. Compared with the MCOTSK model trained with RL only (the last model in Figure 5), the success rate of the model trained with the successive framework was 10% higher, and the median searching steps and distance overhead were similar. This result can demonstrate that the proposed training framework can initialize the action model to a suboptimal parameter setting, and a more robust model can be obtained through further RL training compared with the model trained by RL only.

**Figure 6:**
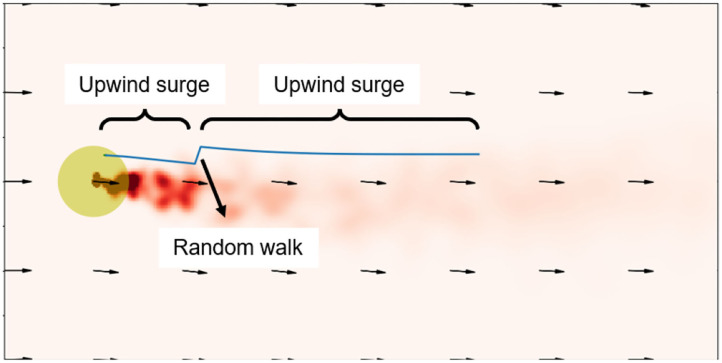
A typical odor source searching trajectory with the MCOTSK model trained with the proposed successive training framework. The blue curves represent the trajectories.

A typical odor source searching trajectory generated by the MCOTSK model trained with the proposed framework was shown in Figure 6. It can be seen that when the robot was in the plumes, it went through an upwind surge path, which is a typical anemotaxis behavior learned in the reinforced training process. When it missed the plumes, it conducted a random walk, which is a behavior inherited from the Fuzzy Lévy Taxis algorithm.

## 4. Discussion

### 4.1. Limitations of the Proposed Framework

Intuitively, the proposed framework can be more time-consuming compared with tuning the controller by supervised training only. In a scenario where edge cases can be ignored and the manually-designed controllers can perform well enough to achieve the goal, the successive training framework may be redundant. Compared with training by RL only, the proposed framework requires a pre-designed controller or some prior knowledge for supervised training, which can be hard in some complex scenarios where existing controllers are not available.

### 4.2. Application Potentials

In this paper, the proposed framework was applied to search for a single odor leakage source. When applied in a scenario where there are multiple odor sources, the proposed training framework can be integrated with various multi-robot odor source searching algorithms (Feng et al., 2019; Wiedemann et al., 2019), that is, to train the robots with supervised learning using the state-action data collected from the existing multi-robot searching algorithm and then to further tune the Actor with RL to learn an optimal action policy.

It is also promising to apply the proposed framework to other robotic problems, e.g., controlling surgical robots (Zhou et al., 2020), industrial manipulators (Su et al., 2020, 2022), and robotic grasping (Deng et al., 2021). The controllers are first initialized with a manually-designed suboptimal controller, and then trained by RL to achieve better performance. Human-robot interactions can also benefit from the proposed framework. The monitored physiological signals (Qi and Su, 2022) and motion signals (Chen et al., 2021b) can serve as the input of the Actor model. The Actor model may be initialized by a generic parameter setting in the supervised training stage. After being trained by RL on each individual user, the robot is expected to cooperate with the user better.

## 5. Conclusion

In this paper, a supervised-reinforced successive training framework for a fuzzy inference system was proposed and applied to a robotic odor source searching problem. The performance evaluation results showed that the proposed method can train the FIS to a suboptimal model through supervised training, and the model trained with further RL can perform better than the model trained with RL only. The results of this paper can inspire researchers to initialize the fuzzy actor model through supervised training using some prior knowledge and then tune a better model with RL.

## Data Availability Statement

The datasets presented in this study can be found in online repositories. The name of the repository and accession number can be found at: GitHub, https://github.com/cxxacxx/MCOTSK.

## Author Contributions

XC contributed to the conception and implementation of the study. YL and CF contributed to supervising the study, reviewing, and revising the manuscript. All authors contributed to the article and approved the submitted version.

## Funding

This work was supported by the National Key R&D Program of China [Grant 2018YFC2001601]; the National Natural Science Foundation of China [Grants U1913205, 62103180, 52175272, and 51805237]; the China Postdoctoral Science Foundation (2021M701577); Guangdong Basic and Applied Basic Research Foundation [Grant 2020B1515120098]; Guangdong Innovative and Entrepreneurial Research Team Program [Grant 2016ZT06G587]; the Science, Technology, and Innovation Commission of Shenzhen Municipality [Grants SGLH20180619172011638, ZDSYS20200811143601004, and KQTD20190929172505711]; the Stable Support Plan Program of Shenzhen Natural Science Fund [Grant 20200925174640002]; Joint Fund of Science & Technology Department of Liaoning Province and State Key Laboratory of Robotics, China [Grant No. 2020-KF-22-03]; Centers for Mechanical Engineering Research and Education at MIT and SUSTech.

## Conflict of Interest

The authors declare that the research was conducted in the absence of any commercial or financial relationships that could be construed as a potential conflict of interest.

## Publisher's Note

All claims expressed in this article are solely those of the authors and do not necessarily represent those of their affiliated organizations, or those of the publisher, the editors and the reviewers. Any product that may be evaluated in this article, or claim that may be made by its manufacturer, is not guaranteed or endorsed by the publisher.
